# Change my body, change my mind: the effects of illusory ownership of an outgroup hand on implicit attitudes toward that outgroup

**DOI:** 10.3389/fpsyg.2013.01016

**Published:** 2014-01-13

**Authors:** Harry Farmer, Lara Maister, Manos Tsakiris

**Affiliations:** ^1^Lab of Action and Body, Department of Psychology, Royal Holloway, University of LondonSurrey, UK; ^2^Institute of Cognitive Neuroscience, University College LondonLondon, UK

**Keywords:** rubber hand illusion, skin color, Prejudice, multisensory processing, social cognition, Implicit Association Test

## Abstract

The effect of multisensory-induced changes on body-ownership and self-awareness using bodily illusions has been well established. More recently, experimental manipulation of bodily illusions have been combined with social cognition tasks to investigate whether changes in body-ownership can in turn change the way we perceive others. For example, experiencing ownership over a dark-skin rubber hand reduces implicit bias against dark-skin groups. Several studies have also shown that processing of skin color and facial features play an important role in judgements of racial typicality and racial categorization independently and in an additive manner. The present study aimed at examining whether using multisensory stimulation to induce feelings of body-ownership over a dark-skin rubber hand would lead to an increase in positive attitudes toward black faces. We here show, that the induced ownership of a body-part of a different skin color affected the participants’ implicit attitudes when processing facial features, in addition to the processing of skin color shown previously. Furthermore, when the levels of pre-existing attitudes toward black people were taken into account, the effect of the rubber hand illusion on the post-stimulation implicit attitudes was only significant for those participants who had a negative initial attitude toward black people, with no significant effects found for those who had positive initial attitudes toward black people. Taken together, our findings corroborate the hypothesis that the representation of the self and its relation to others, as given to us by body-related multisensory processing, is critical in maintaining but also in changing social attitudes.

## INTRODUCTION

Due to its prevalence and importance to society, the formation of people’s attitudes toward members of different racial groups or outgroups in general have been extensively studied by psychological sciences (for a review, see Dunham and Degner, 2010). Research on the formation of stereotypes has shown that people adjust their perception of groups according to their personal experiences with individual members of those groups (Weber and Crocker, 1983; Johnston and Hewstone, 1992; Kunda and Oleson, 1997). A recent longitudinal study showed that liking or disliking of an individual from a particular ethnic group at age 10–12 years predicted general attitudes toward that ethnic group at age 12–13 (Stark et al., 2013). Furthermore, positive experiences with people who have different skin color can lead to a decrease in racial bias (e.g., Kunda and Oleson, 1997; Ensari and Miller, 2002).

More recently there has been an increasing amount of interest in the importance of cultural/racial considerations in cognitive neuroscience (for reviews, see Martïnez Mateo et al., 2012, 2013; Han et al., 2013) which has led to findings demonstrating that racial bias can also exert an effect on lower level bodily aspects of social cognition. Serino et al. (2009) investigated the role of race in the phenomenon of visual remapping of touch (VRT) in which observation of another person being touched leads to more accurate detection of touch on one’s own body. The study found that VRT was modulated by participants’ in-group identification; participants were more accurate in detecting touch when they observed fingers touching a face from the same ethnic group as themselves. Modulations of shared bodily representations based on race have also been observed in studies investigating sensorimotor empathy for pain. Xu et al. (2009) found that the observation of members of a racial outgroup receiving painful stimuli led to less blood oxygen level dependent (BOLD) activation in brain areas involved in pain processing than did the observation of a racial in-group. Avenanti et al. (2010) used transcranial magnetic stimulation (TMS) to observe corticospinal excitability in black and white participants observing a hand of either their own skin color or a different skin color being stabbed with a syringe and found that, while observation of an in-group hand being stabbed led to motor suppression, observation of an outgroup hand being stabbed resulted in motor excitation. Taken together these studies suggest that, as well as affecting cognitive and behavioral level measures, the distinction between racial in-groups and outgroups can also exert an influence on shared body representations.

There has also been a considerable amount of research investigating the factors that can reduce negative implicit attitudes toward racial outgroups. The Implicit Association Test (IAT) is designed to measure attitudes toward other-races that go beyond explicit declarations (Greenwald et al., 1998). Importantly, scores on the race IAT have gained external validation through correlations with behavioral measures of racial bias and racist attitudes in everyday life (McConnell and Leibold, 2001; Richeson and Shelton, 2005; Ziegert and Hanges, 2005; Green et al., 2007; Stepanikova et al., 2011). The wide range of correlations suggests that the IAT provides a valid measure of people’s underlying implicit racial attitudes. Researchers have identified that a wide range of factors can lead to a decrease IAT scores including; training on how to better individuate the faces of people from a different racial group (Lebrecht et al., 2009); being placed in a coalition with members of that racial group (Kurzban et al., 2001); being placed in a situation in which one is subordinate to a member of that racial group (Richeson and Ambady, 2003), having close friends who are members of that racial group (Aberson et al., 2004), behaviorally mimicking a member of that racial group (Inzlicht et al., 2012), and viewing positive exemplars from that racial group (Dasgupta et al., 2001; Ashby Plant et al., 2009).

In line with evidence for a general cognitive bias in favor of automatic positive associations toward the self and self-related stimuli (Greenwald and Banaji, 1995; Mezulis et al., 2004), several of these factors share a common link of increasing the amount of similarity between one’s self and the other racial group (Kurzban et al., 2001; Inzlicht et al., 2012). Indeed several researchers have highlighted the role of self-representation in the processing of ingroup and outgroup relations (Kitayama et al., 1997; Aberson et al., 2000; Schubert and Otten, 2002; Otten, 2003). In general though researchers considering the relationship between the self and social groups have drawn on cognitive theories of self-representation such as the self-concept (Markus and Wurf, 1987). By contrast, recent research in cognitive neuroscience has highlighted the role of body representation in providing the basis for a minimal form of selfhood (e.g., Blanke and Metzinger, 2009; Tsakiris, 2010). In addition a number of recent researchers have argued that our higher level conceptual representations have their evolutionary and developmental basis in lower level sensorimotor representations (Barsalou, 2010; Lakoff, 2012), which suggests that the conceptual representations of the self usually discussed in social cognition may be closely associated with more bodily representation of the self (Farmer and Tsakiris, 2012). This raises the possibility that, through linking the skin color of a racial outgroup to bodily representations of the self, one might be able to alter people’s attitudes toward that racial group.

Is it possible that even a temporary link between one’s bodily self and a body from another racial group may exert an effect on participant’s attitudes toward that racial group? A tentative positive answer to this question was given by Farmer et al. (2012) in what was the first systematic study to investigate whether people can experience a sense of body-ownership for a body of a different skin color, using the rubber hand illusion (RHI, Botvinick and Cohen, 1998) on white participants who observed both a black and a white rubber hand in different conditions. The RHI employs synchronous or asynchronous multisensory stimulation between the participant’s own hidden hand and a fake hand. The integration of synchronous, but not asynchronous, seen and felt touch results in a change in body-ownership (for a review see Tsakiris, 2010). As a measure of racial bias, Farmer et al. (2012) used the race IAT. In two experiments using introspective, behavioral and physiological methods, Farmer et al. (2012) showed that, following synchronous visuotactile (VT) stimulation, participants can experience body-ownership over hands that seem to belong to a different racial group. Interestingly, a baseline measure of implicit racial bias, assessed with the race IAT, did not predict whether participants would experience the RHI, but the overall strength of experienced body-ownership predicted the participants’ post-illusion implicit racial bias with those who experienced a stronger RHI showing a lower bias. These findings suggested that multisensory experiences can override strict ingroup/outgroup distinctions based on skin color, and point to a key role for sensory processing in social cognition. However, because of the within-subjects design of these experiments, it was not possible to specifically address the role of ownership for a black hand, as opposed to a white hand, on implicit associations.

More recently, three studies have used comparable methods to investigate whether a change in self-representations, specifically in the sense of body-ownership, can change implicit attitudes (Banakou et al., 2013; Maister et al., 2013; Peck et al., 2013). Of most relevance for the present study, Maister et al. (2013) used a between subject design to investigate whether the effect of changes in body-ownership over a hand that has a darker skin color would lead to a change in implicit biases against people with dark-skin color. Maister et al. (2013) found a significant relationship between experiencing ownership over the dark-skinned rubber hand and change in IAT scores with those who experienced greater ownership over the dark-skinned rubber hand showing a reduction in racial skin color bias which was not seen with participants who experienced ownership over the light-skinned rubber hand. Importantly, Maister et al. (2013) used the skin color version of the IAT that displays a set of drawings of faces that are identical in the light and dark conditions apart from their skin color and so did not account for the distinctive differences in facial features between white and black people in real life. While the findings of that study support the hypothesis that changes in self-representation can in turn change how the self perceives others, it leaves open the question about the generalization of the effect to the processing of other salient features of racial outgroups.

Several studies have investigated contributions of skin color and facial features to racial categorization and have found evidence that both play an important role (Livingston and Brewer, 2002; Eberhardt et al., 2006; Ronquillo et al., 2007; Stepanova and Strube, 2009; Balas and Nelson, 2010; Balas et al., 2011; Ma and Correll, 2011; Hagiwara et al., 2012; Strom et al., 2012; Ratner et al., 2013). Livingston and Brewer (2002) showed that highly prototypic Black targets (e.g., broad nose, large lips, coarse hair texture, dark-skin color) elicited more prejudice than less prototypic targets. Stepanova and Strube (2009) demonstrated that both skin color and facial features affect judgements of racial typicality and racial categorization independently and in an additive manner, while Hagiwara et al. (2012) showed a similar independent effect of skin color and features on white people’s affective judgments toward black people and Strom et al. (2012) found that white participants were more responsive to facial metrics than to skin color when making racial prototypicality ratings. Underlining the potentially lethal consequences of these findings is evidence that people with both darker skin and more prototypically black facial features are more likely to receive the death sentence (Eberhardt et al., 2006) and that participants and police officers playing a first person shooter computer game are more likely to shoot black avatars with prototypical as opposed to unprototypical features (Ma and Correll, 2011). In addition to these behavioral studies, neuroimaging studies have found that skin color and facial features selectively modulate neural responses to faces. Balas and Nelson (2010) showed participants faces of different races while using EEG to record brain activity and demonstrated that, while the N170 component was modulated only by skin color, the N250 component was sensitive to both skin color and facial features. In a follow up study the same authors showed that the neural signature of the “other-race effect,” in which other-race faces tend to look more alike to observers than faces of their own race (Malpass and Kravitz, 1969; Meissner and Brigham, 2001), only occurs in infants when both skin color and facial features are combined. Given the large amount of evidence for the importance of facial features as well as skin color for perceptions of race it is important to show that the specific effects of experiencing ownership over a hand with a dark-skin color found by Maister et al. (2013) generalize to faces with distinctive black facial features as well as merely a dark-skin color.

To expand on the findings of Maister et al. (2013) and address the limitations of Farmer et al. (2012) the current experiment used a similar between subjects design to Maister et al. (2013) but used a single category version of the race IAT that presents photographs of prototypical white and black faces which allowed for the IAT to directly probe attitudes toward black people as a social group rather than merely about faces with light or dark-skin. Importantly these images are gray scale with no significant difference in luminance between the black and white faces and so the key identifying factors for the racial group of the faces are structural features. The single category black faces IAT (SC-IAT; Karpinski and Steinman, 2006) only required participants to associate either good or bad words with black faces, and thus specifically assesses implicit attitudes toward black individuals, in isolation from attitudes toward white individuals. This enabled the study to focus on the effect of multisensory stimulation on participant’s attitudes toward black people rather than their relative bias between black and white people. White participants experienced either synchronous or asynchronous stimulation over a white or black rubber hand, and their scores in two SC-IATs taken before and after this experience were compared. The asynchronous condition was included to examine whether any effects of the RHI on the SC-IAT were due to synchronous VT-stimulation rather than merely due to visual exposure to a black or white hand. We predicted that participants who experienced ownership of the black hand following synchronous stimulation would become more positive in their attitude toward black people, compared to those in the other conditions.

## MATERIALS AND METHODS

### DESIGN

The study used a between participants design with two factors. The first factor was the synchrony of visual-tactile stimulation (synchronous vs. asynchronous) and the second was the skin color of the rubber hand (black vs. white). The dependent variables were participants’ scores in the SC-IAT for black faces post-VT stimulation and participants’ responses to four statements on a seven point Likert scale taken from Longo et al. (2008). In order to have a baseline measure of participants’ attitudes toward black people participants also completed the same SC-IAT prior to experiencing VT-stimulation.

## PROCEDURE

**FIGURE 1:**
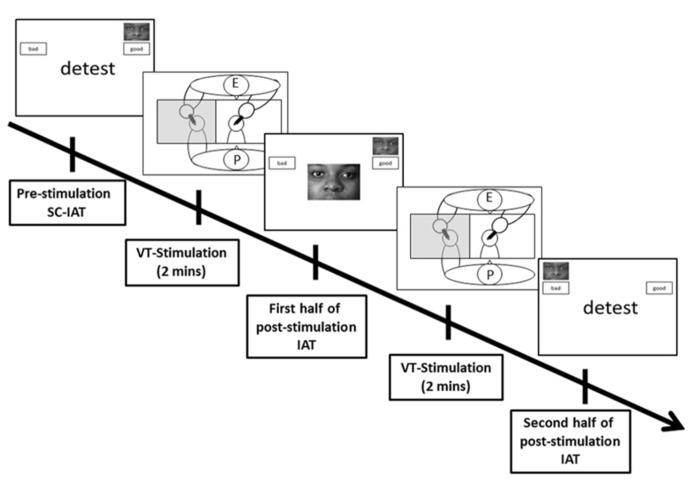
**Structure of experiment (E, experimenter; P, participant)**.

Participants attended one experimental session (see **Figure 1**), in which they first completed a demographic questionnaire. Following this, participants carried out a computer administered SC-IAT, where they categorized words as “good” or “bad” and categorized pictures of black people’s faces as “black” in order to give an initial baseline measure of their implicit attitude toward black people. The associations between stimuli and response key and the order of associations (i.e., positive words and black faces or negative words and black faces) were counterbalanced across participants (Karpinski and Steinman, 2006). The SC-IAT was performed using Presentation^®^ software (Version 16.03, www.neurobs.com). Accuracy and response times were analyzed according to the method used in Karpinski and Steinman (2006) and the resultant scores were adjusted for counterbalancing so that those with a more positive view of black people had positive scores (>0) and those with a more negative view of black people had negative scores (<0).

After completing the SC-IAT participants received stimulation from a paintbrush on their unseen left hand at a frequency of approximately 1 Hz whilst viewing a either a black-skinned or white-skinned rubber hand being stimulated either synchronously or asynchronously with their felt touch. VT stimulation was delivered manually over 2 min with the use of two identical paintbrushes. Both the participant’s left hand and the rubber hand were alternately stimulated on the index, middle and ring fingers from the knuckle to the tip.

Following the 2 min of VT stimulation, participants carried out the first half of the black faces SC-IAT (e.g., the blocks with the associations between black faces and negative words), they then received a further 2 min of VT stimulation before completing the remaining block of the SC-IAT (e.g., black faces and positive words). The order of associations for the two blocks was counterbalanced between participants. Finally participants completed the four-item Ownership questionnaire items which indicated the extent to which they experienced illusory ownership over the rubber hand. They completed these questions twice, once for their experience during the first period of VT stimulation and then again for their experience during the second period of VT stimulation. The questions were presented using Presentation^®^ software (Version 16.03, www.neurobs.com).

### PARTICIPANTS

A total of 148 participants (mean age ± SD: 21 ± 6 years, 43 male) gave their informed consent to participate and were paid for their participation. All participants self-identified as white. The study was approved by the Departmental Ethics Committee, Royal Holloway, University of London.

## RESULTS

### INTROSPECTIVE RATINGS OF THE RHI

**FIGURE 2:**
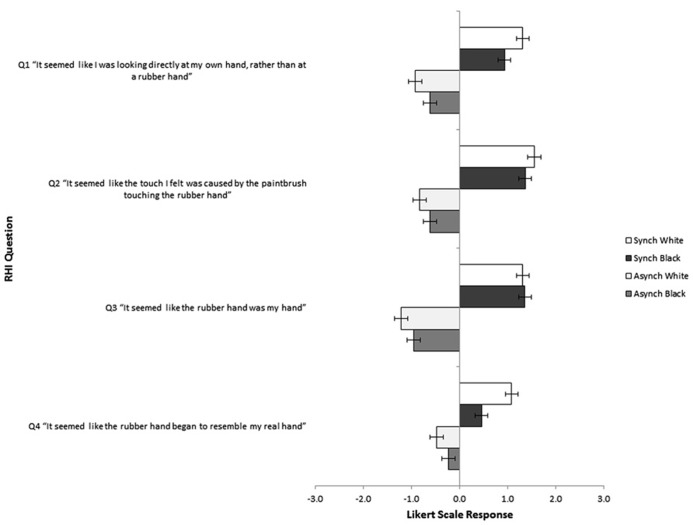
**Mean Likert Scores for each RHI question across each of the four conditions (error bars represent SEM)**.

Participants’ ratings on the four RHI questions were averaged together across the two periods of stroking to produce a mean rating for each question. The data of one participant in the white asynchronous group was lost due to technical error resulting in a total sample of 147 participants. A multivariate analysis of variance (ANOVA) was then run using all of the questions as dependent variables and including synchrony and color as independent variables to establish whether the manipulation of VT stimulation succeeded in generating greater ownership over the rubber hand in the synchronous compared to the asynchronous conditions and to investigate whether the skin color of the rubber hand had any effect on ratings of ownership (see **Figure 2**).

The multivariate analysis of variance (MANOVA) revealed that there was a significant difference between the synchronous and asynchronous groups on question one, “It seemed like I was looking directly at my own hand, rather than at a rubber hand,” *F*(1,143) = 64.32, *p* < 0.001, question two, “It seemed like the touch I felt was caused by the paintbrush touching the rubber hand”, *F*(1,143) = 88.21, *p* < 0.001, question 3 “It seemed like the rubber hand was my hand”, *F*(1,143) = 18.16, *p* < 0.001, and question 4 “It seemed like the rubber hand began to resemble my real hand”, *F*(1,143) = 47.07, *p* < 0.001. There was no significant effect of skin color of the rubber hand or significant interaction between synchrony or skin color for any of the four questions. These results demonstrate that the manipulation of synchronous stimulation was successful in eliciting an illusory sense of body-ownership regardless of the color of the hand.

### PRE-EXISTING IMPLICIT RACIAL BIAS AND EXPERIENCED OWNERSHIP

First, to ensure that there were no significant differences in pre-existing attitudes toward black people between the four groups of participants, a between-subjects ANOVA was carried out on participants’ scores with the pre-stimulation SC-IAT as the dependent variable and synchrony of VT-stimulation (synchronous/asynchronous) and skin color of the rubber hand (black/white) as independent variables. It was found that there were no significant effects of either synchrony of stimulation, *F*(1,144) = 0.49, *p* = 0.487, or of skin color, *F*(1,144) = 0.037, *p* = 0.848, and nor was there a significant interaction between synchrony and skin color, *F*(1,144) = 0.63, *p* = 0.428, indicating that participants across the four groups had comparable scores in the pre-stimulation SC-IAT (**Table 1**).

**Table 1:** Means and standard deviations for SC-IAT and embodiment index in each condition.

	Pre-VT SC-IAT		Embodiment after first session of VT		Embodiment after second session of VT		Post-VT SC-IAT	
	*M*	SD	*M*	SD	*M*	SD	*M*	SD
Synch black	0.01	0.38	0.91	1.32	1.14	1.31	0.05	0.30
Synch white	-0.05	0.35	1.32	1.46	1.31	1.68	-0.01	0.37
Asynch black	0.01	0.44	-0.82	1.51	-0.39	1.71	-0.11	0.28
Asynch white	0.05	0.35	-0.93	1.46	-0.74	1.64	0.10	0.37

We next investigated whether pre-existing implicit attitudes could predict the extent to which participants experienced ownership for the black rubber hand. To do this, participants’ scores in the four introspective questions were averaged together to create an embodiment index, this scale was found to have a high internal consistency (Chronbach’s α = 0.909). Data from participants exposed to the black rubber hand (*n* = 37 for synchronous stimulation, and *n* = 37 for asynchronous stimulation) was then entered in a two-step hierarchical linear regression with the embodiment index as the dependant variable. Pre-stimulation SC-IAT score and synchrony of VT-stimulation (synchronous or asynchronous) were entered as potential predictor variables at the first step, and the interaction between them was entered as a potential predictor variable at the second step. The overall model fit was significant at the first step, *r*^2^ adjusted = 0.28, *F*(2,71) = 15.31, *p* < 0.001. Synchrony of VT-stimulation was the only predictor that explained a significant proportion of the variance [β = 0.53, *t*(71) = 0.53, *p* < 0.001]. Adding the interaction term to the model in Step 2 of the regression did not significantly improve the model fit. Δ*r*^2^ = 0.001, *F*(1,70) = 0.08, *p* = 0.786.

### EFFECT OF SYNCHRONOUS MULTISENSORY STIMULATION ON IMPLICIT ATTITUDES

In order to assess the effect of synchronous multisensory stimulation on implicit attitudes to black people, an analysis of covariance was carried out with participant’s score on the post-stimulation SC-IAT as the dependent variable and two between subjects factors; type of VT-stimulation (synchronous/asynchronous) and skin color of the rubber hand (black/white; **Table 1**). Participant’s pre-stimulation SC-IAT scores were included as a covariate in order to control for participant’s pre-existing attitudes toward black people (as per Huck and Mclean, 1975; Tabachnick and Fidell, 1996).

**FIGURE 3:**
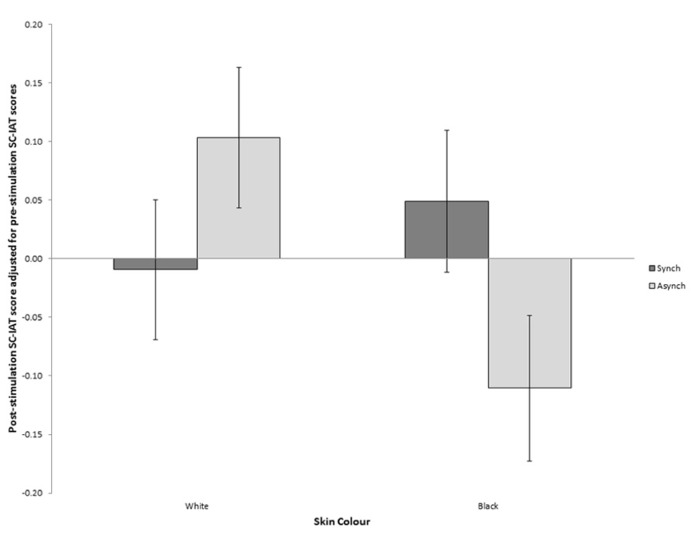
**Post-stimulation IAT scores with pre-stimulation IAT scores covaried out.** Higher values indicate more positive attitudes toward black people. Error bars indicate SEM.

**FIGURE 4:**
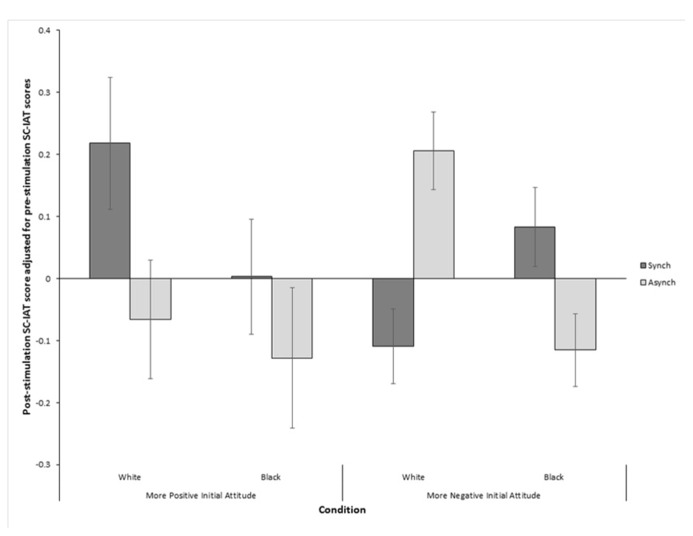
**Post-stimulation IAT scores with pre-stimulation IAT scores covaried out split between those above and below the region of significance (pre-VT-stimulation IAT = 0.077).** Higher values indicate more positive attitudes toward black people. Error bars indicate SEM.

The analysis of covariance (ANCOVA) found no significant main effects of either type of VT stimulation or skin color. However, importantly, a significant interaction between the two factors was found, *F*(1,143) = 6.14, *p* = 0.011, MSE = 0.11 (see **Figure 3**). An interaction was also found between pre VT-stimulation SC-IAT score and synchrony, [*F*(1,140) = 7.87, *p* < 0.006, MSE = 0.1], and between pre VT-stimulation SC-IAT score, skin color and synchrony, [*F*(1,140) = 7.08, *p* = 0.009, MSE = 0.1]. These interactions between the independent variables and the covariate indicated that the homogeneity of regression slopes assumption for ANCOVA had been violated. Therefore, in order to ensure that the results found in the ANCOVA were reliable, the Johnson–Neyman technique recommended by Tabachnick and Fidell (1996) was used to find the regions of significance for the observed effects. It was found that for those participants with a pre-VT stimulation SC-IAT score of above 0.077, indicating more positive attitudes toward black people, there was no significant interaction between VT-stimulation and skin color while the observed effects reported above were significant for those with a pre-VT stimulation SC-IAT score below 0.077 (see **Figure 4**). This indicated that the manipulation was successful in altering attitudes toward black people only if participants originally held relatively negative attitudes toward black people.

In order to further investigate this interaction two additional ANCOVAs were run. The first ANCOVA investigated the effect of VT stimulation on post-stimulation IAT, with pre-stimulation IAT score as the covariate, only on participants in the black rubber hand conditions and revealed a significant effect of synchrony, *F*(1,71) = 5.54, *p* = 0.023, with those who received synchronous stimulation showing a positive adjusted post-stimulation IAT score, indicating an increase in attitudes toward black people while those who received asynchronous stimulation showed a negative adjusted post-stimulation IAT, indicating a decrease in attitudes toward black people. The second ANCOVA included only those participants in the white rubber hand conditions and found no significant effect of synchrony, *F*(1,71) = 1.74, *p* = 0.191, indicating no significant difference in SC-IAT score between those who received synchronous stimulation compared to those who received asynchronous stimulation.

### EFFECT OF FEELING OF BODY-OWNERSHIP ON IMPLICIT ATTITUDES TO BLACK PEOPLE

In order to investigate the effect of experiencing body-ownership over the rubber hand on participants’ implicit attitudes to black people a three-step hierarchical linear regression was carried out with post-stimulation SC-IAT score as the dependant variable. Synchrony of VT-stimulation, embodiment index, and pre-stimulation SC-IAT score were entered as predictor variables in the first step, all two-way interaction terms entered in the second step and the three-way interaction entered as a third step. Due to the finding in the previous analysis that a significant difference in post-stimulation SC-IAT between synchronous and asynchronous groups existed only for participants in the black rubber hand group, only those who saw the black rubber hand were included in the regression. As the embodiment index and synchrony were significantly correlated, *r* = 0.528, *p* < 0.001, we mean-centered the embodiment index for each level of synchrony before adding it to the regression to avoid problems of collinearity. The overall model fit was significant at the first step, *r*^2^ adjusted = 0.085, *F*(1,72) = 3.27, *p* = 0.026. Synchrony of VT-stimulation was the only predictor that explained a significant proportion of the variance [β = 0.15, *t*(71) = 2.19, *p* = 0.032]. Adding the two-way interaction terms to the model in Step 2 of the regression did not significantly improve the model fit, Δ*r*^2^ = 0.001, *F*(3,67) = 0.04, *p* = 0.991, and neither did adding the three-way interaction term in Step 3, Δ*r*^2^ = 0.019, *F*(1,66) = 1.45, *p* = 0.232.

## DISCUSSION

The present study examined whether using multisensory stimulation to induce feelings of body-ownership over the hand of a racial outgroup would lead to an increase in positive attitude toward that racial outgroup. First, and most importantly, we found a significant interaction between the synchrony of VT stimulation and the skin color of the rubber hand. Those participants who received synchronous VT stimulation with the black rubber hand were found to have more positive implicit attitudes toward black people post-stimulation than those who received asynchronous stimulation of the black rubber hand. Second we showed that synchronous multisensory stimulation was successful in eliciting an induced sense of body-ownership over a black rubber hand., Third, we showed that the strength of the experienced ownership was not predicted by pre-existing levels of implicit attitudes against the outgroup, but only by the pattern of stimulation. Finally when the effects of pre-VT stimulation attitudes toward black people was taken into account, the effect of VT stimulation was only significant for those participants who had a low initial attitude toward black people. No significant effects were found for those who had positive initial attitudes toward black people. We discuss these findings in turn.

The most important finding of this study was that of a significant interaction effect between the synchrony of VT stimulation and the skin color of the rubber hand on the post-stimulation implicit racial bias scores. Further analyses demonstrated that this interaction was driven by a significant difference between the synchronous and asynchronous conditions in those who had received VT-stimulation with the black hand but not those who had received it with the white hand. This finding suggests that VT-stimulation modulated the overlap between the black hand and the participant’s own hand. This modulation of overlap was then generalized to the outgroup as a whole, leading to a change in high-level social attitudes, as evidenced by the change in the post SC-IAT. As such this study presents further evidence in line with that of other recent studies (Farmer et al., 2012; Banakou et al., 2013; Maister et al., 2013; Peck et al., 2013) that the plasticity of body representation constitutes a previously unexplored dimension in social cognition processes.

The finding that synchronous VT stimulation is capable of inducing ownership over a hand of a different skin color replicates the previous finding of Farmer et al. (2012) and Maister et al. (2013). Of note is the fact that the study differed from that of Farmer et al. (2012) in finding a main effect of synchrony but not of skin color on body-ownership, whereas Farmer et al. (2012) found a significant difference between ownership scores for the black and white rubber hands. This difference is probably due to the fact that the current study used a between-subject design, while Farmer et al. (2012) employed a within-subject design. Thus, in Farmer et al.’s (2012) study, participants were able to directly compare their experience of ownership over the white and black rubber hands, which is likely to have led them to more closely indicate any perceived difference in feeling of ownership between the two conditions. In support of this hypothesis is the fact that Maister et al. (2013) who used a similar between-subjects design to that employed in the current study, also failed to find any significant effect of skin color on introspective judgements of body-ownership.

In common with the previous findings of both Farmer et al. (2012) and Maister et al. (2013), we found that pre-stimulation attitudes toward the outgroup did not significantly predict feeling of ownership. This result further emphasizes that in the case of multisensory-induced changes in body-ownership, unlike the cases of empathy for pain (Avenanti et al., 2010) and action observation (Gutsell and Inzlicht, 2010), participant’s pre-existing racial bias does not play a significant role in determining the amount of association between self and other. This is an intriguing finding because it suggests that, while processes driven by simulation, such as empathy and action understanding, are affected by factors such as physical and social similarity between self and other, in the case of shared multisensory stimulation these factors are less relevant, possibly because the direct matching of sensory signals between self and other overrides them.

In contrast to Maister et al.’s (2013) study where the change in implicit attitudes was driven by the strength of experienced ownership, the present study did not find a significant effect of the experience of body-ownership over the rubber hand on attitudes toward black people over and above the effect of the synchronicity of stimulation. It is important to note however, that in both Maister et al.’s (2013) study and the study reported here there was a strong association between synchronous VT-stimulation and body-ownership as measured by participants’ responses to RHI questions. This association can be seen by that fact that, defining a mean response to the four RHI questions of greater than zero as constituting an experience of body-ownership, in the current study the vast majority of participants in the synchronous conditions reported experiencing ownership over the rubber hand (84% in total, 84% for the black hand condition). This robust association suggests that, despite the difference in the factor that was found to be most closely linked to changes in attitudes, the results of the current study and that of Maister et al. (2013) are largely in agreement as to the power of multisensory stimulation to change participants’ attitudes toward an outgroup.

Extending the results of Maister et al. (2013), we here used the race IAT that presents photographs of black people’s faces whereas in Maister et al.’s (2013) study the stimuli used were drawings of faces that had been colored to give them either light or dark-skin. As argued in the introduction, several studies have shown that processing of skin color and facial features play an important role in judgements of racial typicality and racial categorization independently and in an additive manner. We here show that the induced ownership of a body-part of different skin color affected the participants’ implicit attitudes when processing facial features, in addition to skin color as shown in previous studies. Thus, the effects of multisensory-induced changes in body-ownership generalize to faces with distinctive black facial features as well as merely a dark-skin color. As highlighted above, a key difference between the current study and that of Maister et al. (2013) is the importance of the strength of body-ownership as shown in Maister et al. (2013) versus the mere fact of a change in body-ownership as shown here. It is possible that in the case of the skin color SC-IAT the strength of the experience of ownership, rather than the fact of whether participants experienced ownership or not, was the key factor in changing implicit attitudes. In the skin color variant of the IAT, the stimuli used do not contain prototypical features of black faces. Instead, the focus is on the skin color, independently of facial characteristics. Skin color can be thought of as a continuous variable that can also account for physical differences within groups or races (Strom et al., 2012). To the extent that participants experienced the dark-skin rubber hand as their own, and the consequent change that this may have had on their body-image (Longo et al., 2009), it is plausible that the actual strength of the illusion would have a greater impact in processing the skin color IAT stimuli as more similar to the self. In the current study, by contrast, where grayscale photographs of black people’s faces were used in the IAT, the more salient nature of the stimuli for race categorization may have meant that the synchrony of stimulation and the consequent change in ownership, but not the strength of this change, was the critical factor in determining changes in attitudes.

A novel finding of the present study is that our experimental manipulation seemed to have an effect on those participants whose prior attitude toward black people was lower while those participants whose attitude toward black people was initially higher were less affected by the manipulation. This finding is important for contextualizing the effects that multisensory-induced changes in self-representations can have on social cognition. While pre-existing levels of implicit biases do not seem to influence whether such multisensory-induced changes can occur for outgroups, we here show that the consequences that such changes have on social cognition are accentuated for people with low pre-existing attitudes toward the outgroup. Given that the IAT seems to be resistant to cognitive strategies or general task demands (Kim, 2003; Steffens, 2004; Fiedler and Bluemke, 2005), the observed changes reinforce the hypothesis that the representation of the self and its relation to others as given to us by multisensory processing is important in maintaining or changing social attitudes. It is also possible however, that the lack of an effect for participants with a high initial attitude might reflect a ceiling effect due to these participants having SC-IAT scores that were too high to be further increased by our manipulation.

Recent studies have utilized virtual reality to investigate the effects of embodiment on implicit social attitudes (Banakou et al., 2013; Peck et al., 2013). Peck et al. (2013) demonstrated that experiencing control of a dark-skinned avatar led to a decrease in implicit racial bias as measured by the race-IAT. These results are convergent with those reported here, despite several methodological differences. First, Peck et al.’s (2013) study involved creating the feeling of body-ownership over a whole body rather than just a hand, indicating that the anatomical location of the body-part embodied does not make a difference to the influence of embodiment on implicit attitudes. Second, whilst the current study used passive multisensory stimulation to induce the feeling of ownership over a black hand, Peck et al. (2013) induced the feeling of ownership over the avatar by creating a sensorimotor experience in which moving one’s own body caused the body of the avatar to move in synchrony. The hypothesis that bias toward an outgroup can be reduced by synchronization between one’s own movements and those of a member of the outgroup has also been supported by the findings of Inzlicht et al. (2012), who have shown that mimicking a member of a racial outgroup can reduce negative attitudes toward that outgroup.

Furthermore, another recent study (Banakou et al., 2013) expanded the investigation of the relationship between embodiment and implicit attitudes by showing that feeling body-ownership over an avatar of a child resulted in a change in implicit attitudes toward children as measured by an IAT. Importantly, the IAT used in Banakou et al. (2013) showed an increase in the association between the self-concept and childlike facial features, indicating that the change in implicit associations seen in that study was due to changes in self-representation. This finding suggests the changes in implicit attitudes toward a racial outgroup found in the current study may also be mediated by changes in self-representation, whereby the self is seen as more similar to members of a racial outgroup.

In conclusion, the present study demonstrates that multisensory stimulation over a hand with the skin color of a racial outgroup can have an effect on cognitive attitudes toward that group. Synchronous stimulation of a black rubber hand led to a significantly more positive attitude toward black people compared to asynchronous stimulation. This finding adds to previous research by demonstrating that by manipulating, through multisensory stimulation, the perceived overlap between one’s own body and a hand of a different racial group it is possible to change social attitudes toward that racial group. Moreover, the current study also expands on previous results showing links between multisensory stimulation and higher level cognition (Farmer et al., 2012; Banakou et al., 2013; Maister et al., 2013; Peck et al., 2013) by suggesting that asynchronous stimulation can influence the perceived closeness between self and other by emphasizing, contrary to the effects of synchronous stimulation, the dissimilarity between one’s own body and that of another.

## Conflict of Interest Statement

The authors declare that the research was conducted in the absence of any commercial or financial relationships that could be construed as a potential conflict of interest.
